# Air Bubble Contact with Endothelial Cells Causes a Calcium-Independent Loss in Mitochondrial Membrane Potential

**DOI:** 10.1371/journal.pone.0047254

**Published:** 2012-10-16

**Authors:** Peter Sobolewski, Judith Kandel, David M. Eckmann

**Affiliations:** 1 Department of Anesthesiology and Critical Care, University of Pennsylvania, Philadelphia, Pennsylvania, United States of America; 2 Department of Bioengineering, University of Pennsylvania, Philadelphia, Pennsylvania, United States of America; University Hospital Freiburg, Germany

## Abstract

**Objective:**

Gas microembolism remains a serious risk associated with surgical procedures and decompression. Despite this, the signaling consequences of air bubbles in the vasculature are poorly understood and there is a lack of pharmacological therapies available. Here, we investigate the mitochondrial consequences of air bubble contact with endothelial cells.

**Methods and Results:**

Human umbilical vein endothelial cells were loaded with an intracellular calcium indicator (Fluo-4) and either a mitochondrial calcium indicator (X-Rhod-1) or mitochondrial membrane potential indicator (TMRM). Contact with 50–150 µm air bubbles induced concurrent rises in intracellular and mitochondrial calcium, followed by a loss of mitochondrial membrane potential. Pre-treating cells with 1 µmol/L ruthenium red, a TRPV family calcium channel blocker, did not protect cells from the mitochondrial depolarization, despite blocking the intracellular calcium response. Mitigating the interactions between the air-liquid interface and the endothelial surface layer with 5% BSA or 0.1% Pluronic F-127 prevented the loss of mitochondrial membrane potential. Finally, inhibiting protein kinase C-α (PKCα), with 5 µmol/L Gö6976, protected cells from mitochondrial depolarization, but did not affect the intracellular calcium response.

**Conclusions:**

Our results indicate that air bubble contact with endothelial cells activates a novel, calcium-independent, PKCα-dependent signaling pathway, which results in mitochondrial depolarization. As a result, mitochondrial dysfunction is likely to be a key contributor to the pathophysiology of gas embolism injury. Further, this connection between the endothelial surface layer and endothelial mitochondria may also play an important role in vascular homeostasis and disease.

## Introduction

Gas embolism occurs when a gas, typically air, enters the vasculature. This can occur during a surgical procedure or as a result of a decompression event [1]. The consequences of gas embolism depend on the size of the gas bubbles and their rate of delivery. Bubbles can cause damage in the microcirculation of any organ, obstruct blood vessels, or even air-lock the heart [2]. Despite greater awareness and improved practice, gas microembolism continues to be a serious risk associated with surgical procedures, particularly those involving cardiopulmonary bypass (CPB), and likely contributes to the incidence of cognitive deficit following such surgeries [3]. In addition, the intravascular formation of gaseous microemboli is known to cause decompression illness [4]. Despite the dangers associated with gas embolism, there is a lack of pharmacological therapies. Further, the “gold standard” therapy, hyperbaric oxygen, is both limited in effectiveness and potentially difficult and dangerous to administer [2].

The vascular sequelae of gas microembolism include endothelial cell damage or dysfunction, as well as platelet activation, complement response, and leukocyte adhesion [1], [4], [5]. This indicates that the endothelium plays a key role in responding to the presence of gas microemboli in the vasculature and mediating their consequences in surrounding tissues. Animal studies have demonstrated that gas microemboli can cause changes in cerebral blood flow and depress neural function, despite not obstructing blood flow [6]. Further, *ex vivo* studies have shown that using a surfactant to reduce the interactions between air bubbles and the endothelium [7] can prevent the loss of vasodilatory function observed in vessels not protected with a surfactant. Collectively, this evidence strongly points to the endothelium as the mediator of deleterious effects of gas embolism.

Our group has developed a platform that enables us to examine the consequences of air microbubble contact with individual endothelial cells in culture. We have established that air bubble contact results in a rapid increase in intracellular calcium and that this increase is associated with cell injury and death [8]. Further, we have examined the mechanism responsible for the intracellular calcium transient, demonstrating that a heparan sulfate proteoglycan (HSPG), syndecan, is the likely sensor [9]. This sensor transduces air bubble contact into an influx of calcium through a TRPV family channel, which, in turn, triggers the release of calcium from intracellular stores via the IP3 pathway [10]. Recent research has established the importance of the interplay between intracellular calcium and mitochondria [11], as well as the role endothelial cell mitochondria play in cardiovascular homeostasis [12]. Thus, in this work, we focus on the mitochondrial consequences of air bubble-endothelial cell contact.

## Materials and Methods

### Cell Culture

As an *in vitro* cell culture model, human umbilical cord vein endothelial cells (HUVEC) were cultured, as described previously [10]. HUVECs (passage 2–5), obtained from Lifeline Cell Technology (Walkersville, MD), were plated in BD Primeria 35 mm cell culture dishes (BD, Franklin Lakes, NJ) approximately 48 hours before planned experiments at a density of ∼3,000 cells/cm^2^ and cultured in VascuLife VEGF Cell Culture Media (Lifeline Cell Technology, Walkersville, MD). Media samples were checked for mycoplasma contamination using MycoAlert Kit (Lonza, Rockland, ME).

### Dye Loading

In all experiments, cells were loaded with the calcium sensitive dye Fluo-4 AM (Invitrogen, Carlsbad, CA) for 20 min at room temperature at a concentration of 1 µmol/L (plus 0.005% Pluronic F-127 (Invitrogen, Carlsbad, CA)), as described previously [10]. In order to assess mitochondrial calcium, cells were loaded with 0.5 µmol/L X-Rhod-1 AM (Invitrogen, Carlsbad, CA) for 60 min at 37°C prior to Fluo-4 loading. In parallel experiments aimed at assessing mitochondrial membrane potential (ΔΨ_m_), cells were loaded with Fluo-4 first, followed by loading with 20 nmol/L TMRM (Invitrogen, Carlsbad, CA) for 60 min at room temperature. All experiments were carried out at room temperature in HBSS, as described previously [10].

### Pharmacological Agents

In order to determine the contributions of various pathways to the mitochondrial response elicited by bubble-cell contact, dye-loaded HUVECs were treated with various pharmacological agents, as described previously [8], [9], [10]. Unless otherwise noted, the agents were handled per manufacturer’s instructions, prepared as 100x stocks in the vehicle noted and were applied individually at room temperature 20 min prior to bubble experiments, with no removal or wash step after exposure. Briefly, 1 µmol/L ruthenium red (Sigma Aldrich, St. Louis, MO) was used to block calcium influx via TRP channels, 5% BSA (Sigma Aldrich, St. Louis, MO) and 0.1% Pluronic F-127 (Invitrogen, Carlsbad, CA) were used as a protein and surfactant treatment respectively, to reduce HSPG-initiated signaling, and 5 µmol/L Gö6976 (LC Laboratories, Woburn, MA) was used as a protein kinase C-α (PKCα) inhibitor [13]. Additional controls included: (1) 2 µmol/L CCCP (Sigma Aldrich, St. Louis, MO), a mitochondrial uncoupler, (2) 5 µg/mL oligomycin (Sigma Aldrich, St. Louis, MO), an ATP synthase inhibitor, and (3) 10 µmol/L ATP (Sigma Aldrich, St. Louis, MO), which causes an intracellular calcium increase.

### Air Bubble-cell Contact Experiments

Air bubble-cell contact experiments were performed as described previously [10]. Drummond Nanoliter micropipettes (Drummond Scientific Co, Broomall, PA) were pulled and ground at a 32.5 degree angle to a diameter of 30–40 µm. A micropipette was then mounted on a Nanoject II injector (Drummond Scientific Co, Broomall, PA), backfilled alternately with HBSS and air, and manipulated with a PPM500 micromanipulator (WPI, Sarasota, FL). Air was expelled using the Nanoject II until a bubble formed at the tip of the micropipette, followed by gradual aspiration until the bubble was 50–150 µm in diameter. The bubble was then manipulated into position 100–200 µm above the perinuclear region of an isolated target endothelial cell and lowered at a rate of ∼5 µm per 3 seconds (z-axis manipulation only) until contact was observed in the phase contrast image capture. Following bubble-cell contact, the bubble was gently lifted and manipulated out of the field of view.

### Microscopy and Image Analysis

Cells were imaged as described previously [10]. Images were captured with a SensiCam QE camera (The Cooke Corp., Romulus, MI) (2×2 binning, 688×520, low-light mode) attached to Olympus IX70 microscope (Olympus, Melville, NY) with an Olympus LUCPlanFL N 40X 0.6NA objective (Olympus, Melville, NY) and Photofluor light source (89 North, Burlington, VT). Computer control of the microscope was facilitated by LUDL programmable filter wheels, shutters, and focus control (Ludl Electronic Products, Hawthorne, NY) using IPL 3.7 software (BD, Rockville, MD). Image stacks were analyzed using ImageJ software (NIH, Bethesda, MD). Fluo-4 signal was analyzed using a nuclear region of interest (ROI), as described previously [10]. The fluorescence ratio (F_R_) for each time point was computed as follows:
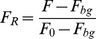
where F is the intensity of the nuclear ROI, F_bg_ is the intensity of a background ROI, and F_0_ is the intensity of the nuclear ROI before bubble contact. For X-Rhod-1 and TMRM fluorescence analysis, stacks were first background corrected and then thresholded to generate mitochondrial ROI. The mean fluorescence of the mitochondrial ROI was then computed at each time point. For X-Rhod-1, F_R_ was computed as a ratio of F, the intensity of the mitochondrial ROI at a given time point, to F_0_, the intensity of the mitochondrial ROI prior to air bubble contact. For normalized TMRM traces, the normalized fluorescence (F^*^) was computed as follows:



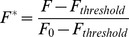
where F is the intensity of the mitochondrial ROI, F_threshold_ is the initial intensity of cytoplasm, used to establish the mitochondrial ROI, and F_0_ is the initial intensity of the mitochondrial ROI. As a control, the fluorescence of TMRM-loaded HUVECs was recorded in the absence of air bubble contact in order to assess photobleaching. In a separate experiment, before and after images of cells were compared in order to assess dye loss from mitochondria over time. An exponential function well approximated the loss of signal observed, and the majority (>80%) was due to photobleaching, with the remainder being leakage of dye out of the mitochondria. SigmaPlot (SysStat Inc., San Jose, CA) was used for data plotting, curve fitting, and statistical analysis.

## Results

### Air Bubble Contact Results in a Rise in Mitochondrial Calcium

**Figure 1:**
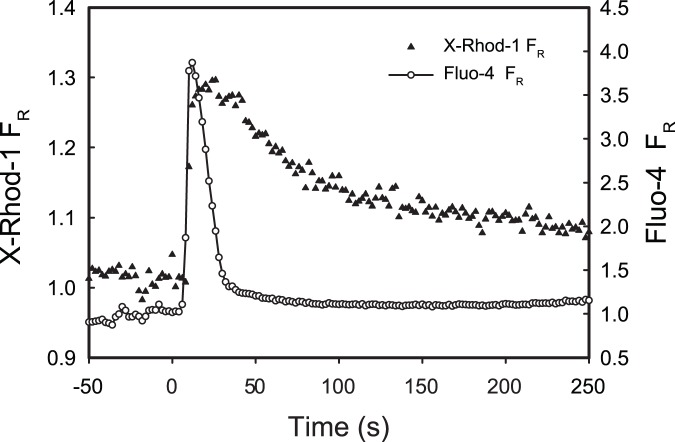
Air bubble contact induces concurrent increases in intracellular and mitochondrial calcium. Representative traces of fluorescence signal (F_R_) obtained from HUVEC loaded with mitochondrial calcium dye X-Rhod-1 (left axis, solid triangles) and intracellular calcium dye Fluo-4 (right axis, open circles) responding to air bubble contact at t = 0.

In order to assess the mitochondrial impact of air bubble contact, HUVECs were stained with X-Rhod-1, a mitochondrial calcium probe. Following contact with an air bubble, individual HUVECs respond with an intracellular calcium transient, which coincided with a rise in mitochondrial calcium (F_R_>1.05) in 11 out of 14 cells. The X-Rhod-1 mean peak F_R_ in the responding cells was 1.15±0.09, which is significantly different from 1 (one-sample t-test, p<.001). See Figure 1 for a representative trace.

### Air Bubble Contact Results in Mitochondrial Depolarization

**Figure 2:**
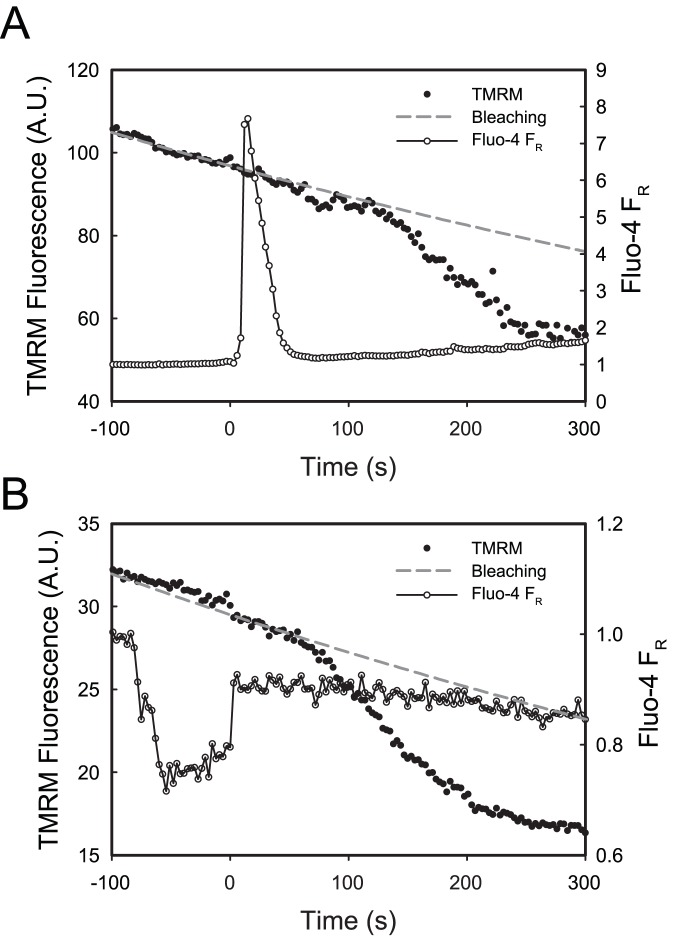
Air bubble contact causes a loss of mitochondrial membrane potential. Representative traces of fluorescence signals obtained from HUVEC loaded with ΔΨ_m_ indicator TMRM (left axis, solid circles) and intracellular calcium dye Fluo-4 (right axis, open circles) responding to air bubble contact at t = 0. Panel **A** was recorded under control conditions, while panel **B** was recorded in the presence of the TRPV channel blocker ruthenium red (1 µmol/L). Dashed grey line represents the expected exponential decay of the TMRM signal due to photobleaching.

**Figure 3:**
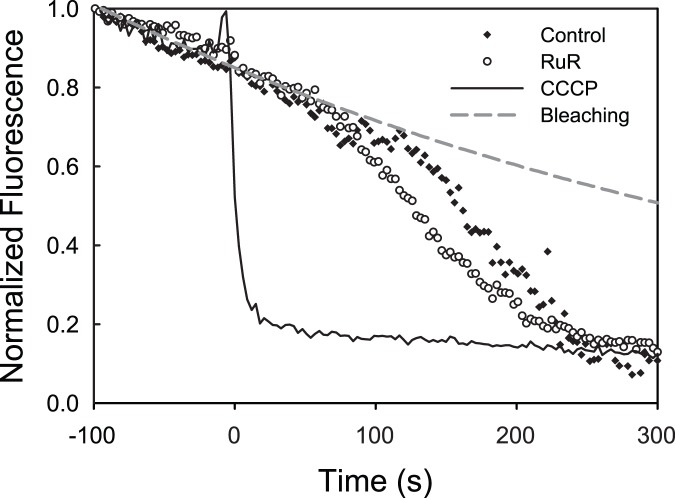
Normalized traces of air bubble contact-induced loss of mitochondrial membrane potential, compared to treatment with CCCP, a mitochondrial uncoupler. Normalized representative traces of fluorescence signal (from Figure 2) obtained from HUVEC loaded with ΔΨ_m_ indicator TMRM responding to air bubble contact under control conditions (solid diamonds) or following pre-treatment with 1 µmol/L ruthenium red (open circles). Solid line represents response to treatment with 2 µmol/L CCCP. Dashed grey line represents the expected exponential decay of the signal, primarily due to photobleaching.

**Figure 4:**
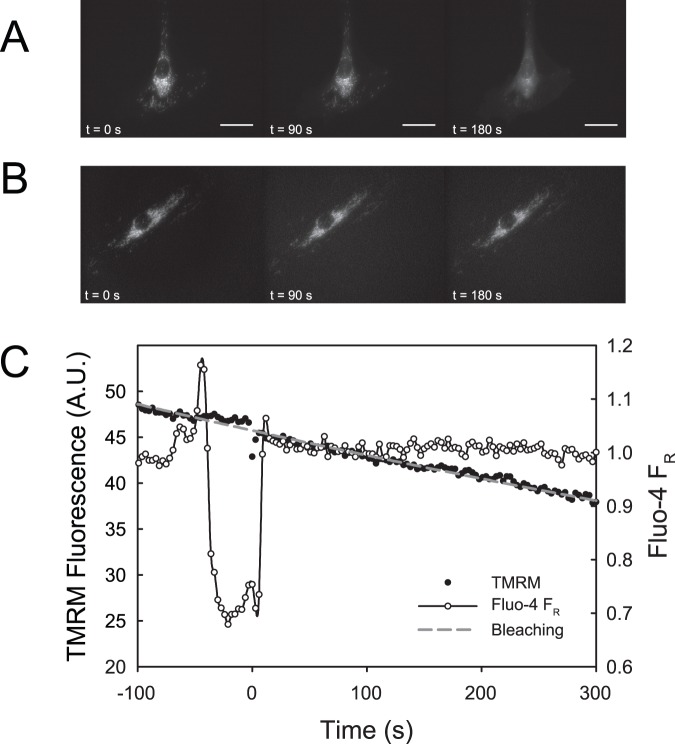
Pre-treatment with 5% BSA prevents air bubble-induced mitochondrial depolarization. A ) Montage of fluorescent photomicrographs of TMRM loaded HUVEC illustrating the progression of ΔΨ_m_ loss following air bubble contact under control conditions. The time-stamps indicate time relative to bubble contact and the scale bar indicates 20 µm. The reduced background in the first frame is the result of the presence of the air bubble, which displaces the buffer (which contributes background fluorescence). **B**) Montage of fluorescent photomicrographs of TMRM loaded HUVEC treated with 5% BSA prior to air bubble contact illustrating maintained ΔΨ_m_. The time-stamps indicate time relative to bubble contact and the scale bar indicates 20 µm. **C**) Corresponding traces of fluorescence signals obtained from HUVEC loaded with ΔΨ_m_ indicator TMRM (left axis, solid circles) and intracellular calcium dye Fluo-4 (right axis, open circles) responding to air bubble contact at t = 0 following pre-treatment with 5% BSA. Dashed grey line represents the expected exponential decay of the signal, primarily due to photobleaching.

In order to determine whether air bubble contact has an effect on mitochondrial membrane potential (ΔΨ_m_), HUVECs were stained with the ΔΨ_m_ indicator dye TMRM. In 15 out of 17 cells, air bubble contact resulted in mitochondrial depolarization, which began 1 to 2 minutes after contact and lasted for 2 to 3 minutes. See Figure 2A for a representative trace. Treating HUVECs with 2 µmol/L CCCP (a mitochondrial uncoupler) resulted in a similar TMRM signal decrease as air bubble contact, indicating complete mitochondrial depolarization. However, CCCP-induced depolarizations were much more rapid, occurring within 30 s. See Figure 3 for normalized traces. Additionally, see Figure 4A for a montage of epifluorescence photomicrographs corresponding to the trace in Figure 2A. Note the decrease in mitochondrial fluorescence and corresponding increase in fluorescence of the cell body, indicating the decompartmentalization of the TMRM dye and loss of ΔΨ_m_.

### Blocking Calcium Influx is Not Sufficient in Preventing Mitochondrial Depolarization

We have previously identified calcium influx via TRPV family channel as the trigger for the intracellular calcium response to air bubble contact [10]. To prevent this calcium response, we pre-treated HUVECs with TRPV family channel blocker, ruthenium red (1 µmol/L). Despite preventing the air bubble-induced intracellular calcium transient, ruthenium red pre-treatment did not prevent mitochondrial depolarization in 5 out of 6 cells. See Figure 2B for a representative trace. Note that the downward deflection in the Fluo-4 F_R_ trace is the result of scattering and refraction caused by the presence of the air bubble in close proximity to the focal plane, as well as displacement of the buffer (which contributes background fluorescence). The degree of depolarization in the presence of ruthenium red was similar to control conditions, as well as to treatment 2 µmol/L CCCP, indicating complete mitochondrial depolarization (see Figure 3 for normalized traces).

### Protein or Surfactant Treatment Prevents Air Bubble-induced Mitochondrial Depolarization

We have previously demonstrated that protein (5% BSA) and surfactant (0.1% Pluronic F-127 or 10% Oxycyte) can mitigate the endothelial cell response to air bubble contact [8], [9]. Repeating the TMRM experiments in the presence of 5% BSA prevented air bubble-induced mitochondrial depolarization and intracellular calcium transients in 6 out of 7 cells. Similar results were obtained in the presence of 0.1% Pluronic F-127, with mitochondrial depolarization being prevented in 7 out of 10 cells and intracellular calcium transients being blocked in all 10. A comparison of representative epifluorescence photomicrographs from control and 5% BSA treated HUVECs is provided in Figures 4A and B respectively, while Figure 4C shows the trace corresponding to the images in Figure 4B. Note that the deflection in the Fluo-4 F_R_ trace is the result of scattering and refraction caused by the presence of the air bubble in close proximity to the focal plane, as well as displacement of the buffer (which contributes background fluorescence).

### PKCα Inhibition Prevents Air Bubble-induced Mitochondrial Depolarization

**Figure 5:**
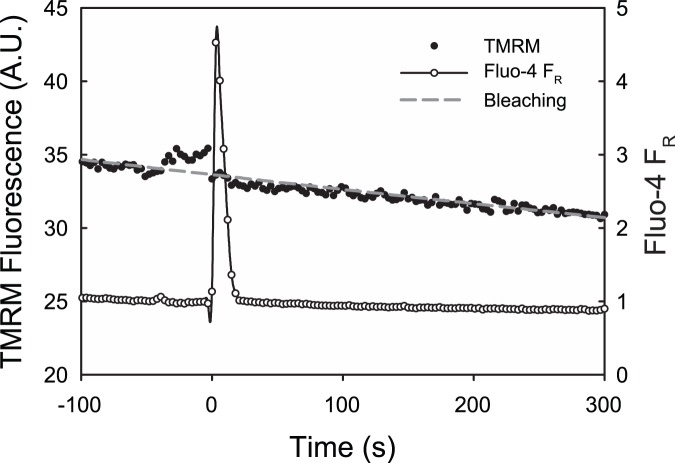
Pre-treatment with PKCα inhibitor Gö6976 prevents air bubble-induced mitochondrial depolarization. Representative trace of fluorescence signals obtained from HUVEC loaded with ΔΨ_m_ indicator TMRM (left axis, solid circles) and intracellular calcium dye Fluo-4 (right axis, open circles) responding to air bubble contact at t = 0 following pre-treatment with 5 µmol/L Gö6976. Dashed grey line represents the expected exponential decay of the signal, primarily due to photobleaching.

We have previously demonstrated that syndecan, an HSPG, is the likely sensor of air bubble contact [9]. Syndecan is known to interact with PKCα and facilitate its activation [14], and PKCα activation has been observed to cause a reduction in ΔΨ_m_ [15]. To test whether PKCα activation is involved in the mitochondrial depolarization following bubble contact, we pre-treated Fluo-4/TMRM-stained HUVECs with 5 µmol/L Gö6976, a PKCα inhibitor. This treatment did not block the intracellular calcium response to air bubble contact, but did prevent mitochondrial depolarization in 4 out of 6 cells. See Figure 5 for a representative trace.

## Discussion

We have previously established that air bubble contact with endothelial cells results in large intracellular calcium transients [8], [9], [10] and that these transients are associated with cell injury and death [8]. Endothelial cell mitochondria play an important role in vascular homeostasis and disease, by regulating intracellular calcium and being able to initiate cell death via the intrinsic apoptosis pathway [12]. The mitochondrial membrane potential (ΔΨ_m_) facilitates the movement of ions, including calcium [11]. As cytosolic calcium concentration rises, calcium from the cytosol enters the mitochondria via a uniporter and can be extruded from the mitochondrial matrix via sodium-calcium exchange, which is coupled to sodium-H^+^ exchange. This exchange process results in a reduction in ΔΨ_m_. If the calcium exchange and buffering capacity is exceeded, the consequences include a loss of ΔΨ_m_, uncoupling, and the loss of mitochondrial contents via opening of the mitochondrial permeability transition pore (mPTP). The opening of the mPTP leads to the release of caspase activating factors, such as cytochrome *c*, which triggers the intrinsic pathway of apoptosis, although cytochrome *c* can also be released in an mPTP-independent fashion [12].

We hypothesized that these cytosolic calcium transients could be sufficient to overload mitochondria and result in mitochondrial depolarization and dysfunction, leading to the cell injury and death we observed previously [8]. In order to determine whether air bubble contact results in a rise in mitochondrial calcium, we loaded HUVECs with the mitochondrial calcium dye, X-Rhod-1, in addition to the intracellular calcium indicator Fluo-4. As we had anticipated, air bubble contact in cells loaded with both dyes resulted in large intracellular calcium transients, as well as concurrent rises in mitochondrial calcium (Figure 1). Given the interplay between mitochondrial calcium and ΔΨ_m_, we next decided to determine whether air bubble contact causes a loss of ΔΨ_m_, which indicates mitochondrial dysfunction. We loaded HUVECs first with Fluo-4, followed by TMRM, a fluorescent ΔΨ_m_ indicator. Due to the overlapping fluorescence spectra of X-Rhod-1 and TMRM, we were not able to assess ΔΨ_m_ at the same time as mitochondrial calcium; however, in parallel experiments, air bubble contact with TMRM and Fluo-4 loaded HUVECs resulted in a marked reduction in ΔΨ_m_ (Figure 2A and 4A). Comparison of bubble contact to treatment with the mitochondrial uncoupler CCCP indicates that the mitochondrial depolarization was complete (Figure 3). Interestingly, there is a substantial lag between the intracellular and mitochondrial calcium increase and the loss of ΔΨ_m_, suggesting that an alternate, calcium independent pathway may be responsible for the loss of ΔΨ_m_. These experiments associate intracellular calcium increase, mitochondrial calcium increase, and mitochondrial dysfunction following air bubble contact.

Further evidence for the calcium independence of the signaling pathway leading to the loss of ΔΨ_m_ is demonstrated by our ruthenium red experiments. We previously demonstrated that the air bubble contact-induced, large intracellular calcium transients are the direct result of IP3-dependent release of intracellular calcium, but that the triggering mechanism is actually a calcium influx via a TRPV family channel [9], [10]. Thus, if the intracellular calcium transient were responsible for the mitochondrial depolarization, blocking the cytosolic calcium transients by treating cells with ruthenium red, a TRPV channel blocker, should prevent the reduction in ΔΨ_m_ caused by air bubble contact. Surprisingly, while ruthenium red did block the intracellular calcium response as we have previously documented [10], it did not prevent the reduction in ΔΨ_m_ in 5 out of 6 cells (Figure 2B). The mitochondrial depolarization was of similar magnitude as that observed in control cells or cells treated with the CCCP, a mitochondrial uncoupler (Figure 3). Thus, contrary to our initial hypothesis, intracellular calcium transients are not necessary for air bubble-induced mitochondrial dysfunction to occur. This finding suggests that air bubble-cell contact causes the activation of an alternative, calcium-independent signaling pathway with adverse mitochondrial consequences.

**Figure 6:**
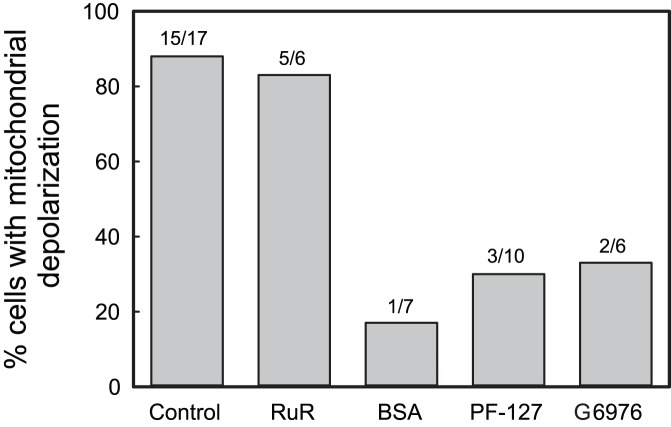
Summary of pharmacological interventions. Percentage of HUVECs exhibiting loss of ΔΨ_m_ following air bubble contact under control conditions or following pre-treatment with 1 µmol/L ruthenium red (RuR), 5% BSA, 0.1% Pluronic F-127 (PF-127), or 5 µmol/L Gö6976 (G6976).

Air bubble contact with endothelial cells results in an interaction between the air-liquid interface and the endothelial surface layer (ESL), in addition to any mechanical deformation of the cells themselves. It is well established that proteins can adsorb to air-water interfaces [16], [17] and that this adsorption can cause conformational changes, including protein denaturation [18], [19]. Many proteins may be involved in the case of air bubble-endothelial cell contact, as the endothelial cell surface is rich in both membrane bound and adsorbed biomolecules which comprise the ESL [20]. Of particular interest are the membrane bound proteins, such as HSPGs, which have signaling capabilities via transmembrane/cytoplasmic domains [21], and the glycocalyx glycosoaminoglycans (GAGs) connected to them. In prior work done by our group, we identified the GAG heparin sulfate (HS) and the HSPG syndecan as responsible for transducing air bubble contact into calcium influx via a TRPV family channel [9]. Further, the incidence and magnitude of intracellular calcium transients could be reduced via protein or surfactant pre-treatment, by minimizing the interaction between the air-liquid interface and the ESL [9]. To examine whether a similar mechanism was responsible for mitochondrial depolarization, we treated HUVECs with 5% BSA or 0.1% Pluronic F-127 prior to air bubble contact. Mechanistically, surfactants rapidly populate the air-liquid interface and can displace, and then block, protein adsorption [16], [17]. Similarly, proteins present in the liquid can interact with the bubble by rapidly adsorbing to the bubble surface [22], as well as with the glycocalyx [23], resulting in a thickening of the endothelial surface layer [20]. Both of these effects serve to minimize direct interactions between the air-liquid interface and ESL components, mitigating signal transduction. In contrast to ruthenium red pre-treatment, which eliminated the intracellular calcium transient but not the loss of ΔΨ_m_, 5% BSA treatment blocked both the intracellular calcium response and the loss of ΔΨ_m_ in all but 1 cell, while 0.1% Pluronic F-127 treatment blocked the intracellular calcium response in all cells and the loss of ΔΨ_m_ in 7 out of 10 (Figure 6). Further, by preventing air bubble-induced mitochondrial dysfunction, BSA and Pluronic F-127 may in turn prevent the activation of apoptosis. This may explain our earlier findings that 5% BSA or 0.1% Pluronic F-127 treatment resulted in a marked protection from cell death following air bubble contact [8].

Recent research has established the importance of syndecans as signaling molecules [24], including our own work implicating syndecan in the mechanotransduction of air bubble contact [9]. Here we hypothesize that syndecan-4 activation of PKCα [14] may be responsible for the mitochondrial depolarization we observe following air bubble contact. A recent study has implicated both syndecan-4 and PKCα in pro-apoptotic signaling [25], while in another study, PKCα activation was observed to cause translocation of PKCα to mitochondria and subsequent reduction in ΔΨ_m_ [15] We attempted to confirm the involvement of syndecan in the mitochondrial response to air bubble contact using PMA to induce syndecan shedding, as we had done previously [9]. However, the PMA treatment resulted in mitochondrial depolarization (data not shown), likely via PKCα activation [15]. As an alternate approach, we utilized Gö6976, an indolocarbazole which inhibits PKCα and PKCβ, but not PKCδ [13]. In contrast to BSA or Pluronic F-127 treatments, which blocked both the intracellular calcium response and the mitochondrial depolarization, Gö6976 treatment only prevented the mitochondrial depolarization (Figure 5). This finding confirms that the air bubble-induced intracellular calcium response and mitochondrial depolarization occur via different, parallel pathways. The mitochondrial depolarization occurs via a calcium independent, PKCα dependent pathway, which is likely activated by syndecan-4. The cytoplasmic V domain of syndecan-4 contains a PIP_2_ binding site, as well as another binding site which can bind α-actinin (an actin bundling protein) and PKCα in a competitive manner [14]. Importantly, these interactions are regulated by phosphorylation at Ser^183^ [26]. When dephosphorylated, syndecan-4 binds to PIP_2_, which facilitates dimer/oligomerization of syndecan-4, PKCα binding, and activation. Meanwhile, Ser^183^ phosphorylation by PKCδ reduces PIP_2_ binding [14] and may increase α-actinin binding [24]. We hypothesize that the transient interaction between the air-liquid interface and the ESL may facilitate the oligomerization of syndecan-4, which is required for and potentiates PKCα activation, but not stabilize it to the extent that occurs in focal adhesions [27]. Thus, PKCα can be activated, but then released, enabling it to translocate to the mitochondria, ultimately causing mitochondrial depolarization.

In conclusion, we have demonstrated that air bubble-endothelial cell contact causes mitochondrial dysfunction. We have identified the existence of a novel, ESL-transduced signaling pathway by which this occurs. While further studies will be needed to elucidate the exact mechanism responsible for inducing mitochondrial dysfunction, we demonstrate here that the process is calcium independent and PKCα dependent. Additionally, our findings further support the concept of surfactant therapy for gas embolism [28], because surfactant treatment can ameliorate the mitochondrial effects of air bubble contact. Finally, a signaling pathway linking the ESL and endothelial mitochondria may also play a broader role in vascular homeostasis and disease, because the glycocalyx [29] and HSPGs in particular [30] have been postulated to play an important role in transducing shear stress.
